# MoDeSuS: A Machine Learning Tool for Selection of Molecular Descriptors in QSAR Studies Applied to Molecular Informatics

**DOI:** 10.1155/2019/2905203

**Published:** 2019-02-17

**Authors:** María Jimena Martínez, Marina Razuc, Ignacio Ponzoni

**Affiliations:** ^1^Instituto de Ciencias e Ingeniería de la Computación (UNS-CONICET), Departamento de Ciencias e Ingeniería de la Computación, Universidad Nacional del Sur (UNS), CP 8000, Bahía Blanca, Argentina; ^2^Comisión de Investigaciones Científicas de la Provincia de Buenos Aires (CIC), Calle 526 between 10 and 11, CP 1900, La Plata, Argentina

## Abstract

The selection of the most relevant molecular descriptors to describe a target variable in the context of QSAR (Quantitative Structure-Activity Relationship) modelling is a challenging combinatorial optimization problem. In this paper, a novel software tool for addressing this task in the context of regression and classification modelling is presented. The methodology that implements the tool is organized into two phases. The first phase uses a multiobjective evolutionary technique to perform the selection of subsets of descriptors. The second phase performs an external validation of the chosen descriptors subsets in order to improve reliability. The tool functionalities have been illustrated through a case study for the estimation of the ready biodegradation property as an example of classification QSAR modelling. The results obtained show the usefulness and potential of this novel software tool that aims to reduce the time and costs of development in the drug discovery process.

## 1. Introduction

Molecular Informatics is an emerging interdisciplinary that addresses mathematical and computational problems, related to molecule-based information encoding and processing, oriented to the discovery of new knowledge in several fields as pharmacology, material engineering, or environmental sciences [1–4] In particular, Quantitative Structure-Activity Relationships (QSAR) modelling constitutes active area of research in Molecular Informatics. QSAR models have been proposed in order to estimate several biological properties, such as activity [5, 6] or ADMET properties [7, 8], providing relevant information to support drug discovery projects [9, 10]. The advantages of having QSAR models for drug design are numerous: reduction of the time spent during the discovery phase, reduction of economic and material resources required due to a decrease in the number of traditional tests, reduction of animal testing, among others.

During the last years, the sizes of chemical compound databases have expanded considerably. However, this abundance in the availability of data has not been able to avoid the growth of the failure rate in the preclinical phases and the “attrition rate”, that measure the proportion of candidate compounds to constitute new drugs that are discarded during the different phases of a drug design project [11]. In this sense, having accurate QSAR models can help improve these issues.

QSAR studies require the codification of the chemical structure of compounds by a diversity of molecular descriptors [12], such as constitutional, geometrical, functional groups, topological, thermodynamic, and quantum mechanical. Currently, the development of new cheminformatics software allows calculating thousands of molecular descriptors, but usually only a small subset of these descriptors brings necessary information for obtaining the QSAR model of interest [13]. Subsequently, the precision of these models rests on the correct selection of molecular descriptors used during the QSAR model generation [14].

Several machine learning approaches have been proposed for addressing the selection of molecular descriptors in an automatic [15] or semiautomatic fashion [16], which are usually known in computer science as* feature selection methods*. This complex task can be characterized as a multiobjective combinatorial optimization problem, where several subsets of molecular descriptors are alternatively selected and evaluated in terms of different optimization criteria [17]. In general, subsets which achieve high predictive accuracy and low cardinality are usually preferred by QSAR model designers [14]. Multiobjective techniques have various uses in QSAR, such as (1) multiobjective optimization of drugs [18–22], (2) development of multitarget models [23–25], (3) multiscale modelling [26–28], (4) chemical, preclinical, omics, and epidemiological data fusion [29–33], and (5) multiobjective feature selection [34–36]. Some authors have worked on the four first applications mentioned before, for instance, the multiobjective method Perturbation-Theory Machine Learning (PTML) with applications in cheminformatics, nanotechnology, omics, and pharmacoepidemiology [37]. However, there are not many reports of multiobjective approaches for feature selection in this area.

In Soto et al. [38], a multiobjective feature selection method for prioritization of molecular descriptors subsets in QSAR studies is presented. The proposed algorithm is organized as a two-phase methodology. The first one makes use of a multiobjective evolutionary technique that yields interesting advantages compared to monoobjective feature selection methods [39]. The second phase enables refining and improving the confidence in the chosen descriptors subsets. This methodology allows the selection of subsets when a large number of descriptors are involved and it is also suitable for linear and nonlinear QSAR regression models.

Later, a software tool, named as DELPHOS, was implemented based on this two-phase methodology [40]. This tool has been successfully applied in the development of regression models for predicting a wide variety of properties [41–44]. Nevertheless, some drawbacks and limitations are present in DELPHOS. First of all, the computational methodology behind this software has only been designed for selecting descriptors in the context of QSAR regression models, but feature selection for QSAR classification models has not been considered. Another drawback is related to the evolution of the state-of-art of machine learning methods, because during the last decade new promising approaches have been proposed in the literature. In this sense, the same machine learning methods used in different steps of the two-phase feature selection methodology can be updated to more robust methods such as Random Forest [45, 46] or Random Committee [47] methods. Finally, DELPHOS was codified using proprietary software [48]. Lamentably, this decision presented some disadvantages because new versions of the proprietary software are not fully compatible with the version used for programming the tool, and the use of DELPHOS code is limited to license holders of this proprietary software.

In this paper, a novel software tool, called* MoDeSuS* (*for Molecular Descriptors Subsets Selection*), is presented. This new feature selection tool was designed in order to address all DELPHOS limitations explained above, which constitutes the main contribution of this work. Additionally, a case study for QSAR modelling in the context of classification problems for drug properties estimation is presented for illustrating the software application in pharmacology. Finally, an integral view of all functionalities and advantages of using MoDeSuS for drug design projects is discussed in conclusion of this article.

## 2. Materials and Methods

### 2.1. QSAR Modelling and Feature Selection

**Figure 1:**
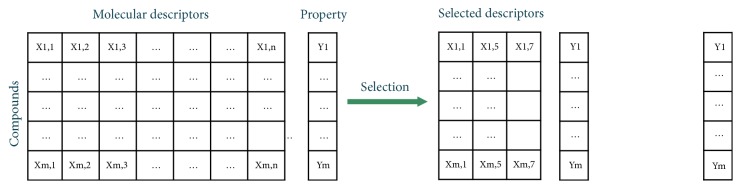
Representative scheme of descriptor selection process.

QSAR models establish relationships between some structural characteristics of a chemical compound and a specific physicochemical or biological property of interest [1]. QSAR models can be inferred through supervised learning processes, using compounds databases for which the experimental values of the target variable to be modelled are already known. Usually, the inference of these models is done using machine learning strategies that require the addressing of several computational subproblems [49]. Among them is the process of selecting the most relevant molecular descriptors for the modelling of the target variable [14], illustrated in Figure 1, which is a particular case of the problem of* feature selection*. A database containing compounds, molecular descriptors, and a target variable to be modelled is required to carry out this combinatorial process of descriptor selection. Then, a machine learning strategy is applied to select and evaluate different subsets of descriptors in order to identify a reduced group of them.

### 2.2. Feature Selection as Multiobjective Combinatorial Optimization

**Figure 2:**
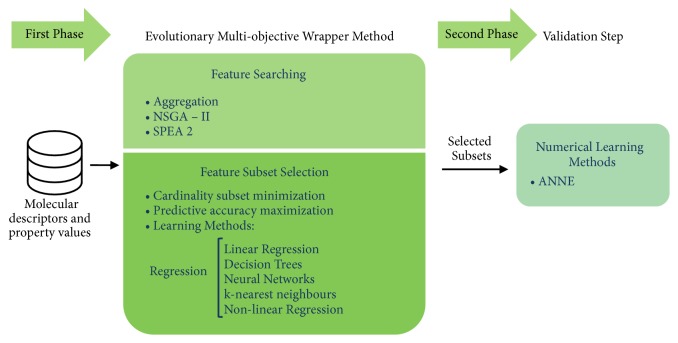
DELPHOS two-phase feature selection methodology.

The methodology presented in [38] implements a multiobjective optimization method, based on two phases, in order to identify subsets of descriptors relevant to the target variable. The first phase makes use of multiobjective combinatorial optimization and acts as a coarse selector of descriptors subsets, while the second phase performs an accurate evaluation of the subsets of the last general selection.  Figure 2 shows an overview of the two-phase methodology.

*First phase *is as follows: as shown in Figure 2, a wrapper method can be divided into two parts: Feature Searching and Feature Subset Evaluation. The first is responsible for conducting the combinatorial search between different selections of feasible subsets. In this sense, binary vectors were used to represent individuals. Each vector has* n* components, that is, one bit for each available descriptor. A nonzero value in the i^th^ bit position of the vector indicates that the i^th^ descriptor is chosen within the selection of the individual. In contrary, a null value in the i^th^ bit position of the vector indicates that the i^th^ descriptor is not chosen within the selection of the individual. The second part evaluates the usefulness of the selected subset and in this way guides the Feature Searching in the selection of the most relevant descriptors. In this sense, in the Feature Searching two different approaches were applied: aggregation and Pareto. In the first one, the searching is guided by a formula that combines two objectives in order to evaluate the relevance of each subset of descriptors. The first objective function F1 calculates the number of selected descriptors. The second objective function F2 estimates the predictive accuracy of a method using the selected descriptors; more precisely, this function computes the mean square error of prediction applied to a set of compounds not used for training. In particular, aggregation allows multiple objectives to be combined into a single fitness function. From the F1 and F2 functions, the following proposed aggregation formula arises:

(1) $$\begin{array}{c} F_{AG}=\alpha F_{2}+\left(\;1-\alpha \;\right)F_{2}\frac{F_{1}}{p_{m}} \end{array}$$

In (1), *α* is a weighting parameter for each objective with possible values in the interval [0,1], and *p*_*m*_ is a parameter that represents the maxima cardinality of a subset. On the other hand, the Pareto based techniques (NSGA II and SPEA 2) optimizes the objectives separately according to the concept of dominance. Dominance is a partial order that can be established between vectors defined over a space R^k^, where k is the number of objectives to be optimized. In this case, since we have two functions (F1 and F2), the defined space is R^2^. From the definition of dominance, the term Pareto front is derived, which is the set of optimal solutions within the problem space and the nondominated front contains the solutions found that are not dominated by any other solution.

*Second phase* is as follows: after applying a combination of any feature search method and evaluation for the multiobjective wrapper, a front of nondominated individuals of each execution is formed. All nondominated subsets obtained in the same run are treated as the set of the most interesting solutions found by the wrapper in that run. Each subset of descriptors in the front is evaluated by a validation method; in particular a set of artificial neural networks (ANNE) was used.

### 2.3. Machine Learning Methods for Regression and Classification

Linear regression is a mathematical method that models the relationship between an output variable (y), independent variables (xi), and a random error term (*ε*). In the case of simple regression, we have a single independent variable x, x *∈* R. Multiple linear regression is an extension of the simple one where the independent variable x is a vector, such that x *∈* Rn.

Regression trees are decisions trees applied to regression problems. In this sense, each internal node of the tree represents a condition (for example, if the feature value exceeds or not a certain threshold) and each leaf denotes the function of regression to be used. The coefficients of this regression function will be the features that guided the path to that leaf. Further, provide a mechanism for pruning and thus keep the minimum height of the tree avoiding overfitting.

Neural Networks (multiperceptron) method classifies instances through backpropagation. This network can be monitored and modified during training time. The nodes in the network are all sigmoid (except for when the class is numeric in which case the output nodes become unthresholded linear units).

k-nearest neighbours method consists of assigning the instance to classify the majority label among the nearest k neighbours. The measure most commonly used to measure closeness is the Euclidean distance.

Random Forest generates a forest of random trees [46]. This arbitrary set of independent decision trees is tested on random datasets that have the same number of variables selected at random, performing no pruning. Also, it has an option to allow estimation of class probabilities (or target mean in the regression case) based on a hold-out set (backfitting).

Random Committee builds an ensemble of randomized base classifiers. Each base classifier is built using a different random seed. The final predict value is a straight average of the predictions generated by the individual base classifiers.

In decision trees, the data is recursively divided into smaller sets with binary partitions. In each iteration of the method, different partitions are evaluated (evaluating the whole dataset) and the best one is chosen. The division of the data generates as output of the method a tree structure, where each node represents one of the input variables. Each leaf node in the tree represents a value of the destination variable. That is, the predicted value of the destination variable is obtained by the path traveled from the root to a leaf of the tree.

### 2.4. Molecular Information Datasets for Case Studies

The dataset used for the classification case study was extracted from [50]. It consists of 1725 molecules, 1480 molecular descriptors calculated by using Dragon [51], and the experimental values of the target variable: ready biodegradation. These values have been reported after performing a test that measures the biochemical oxygen demand (BOD). In this sense, chemical compounds with a BOD value greater than 60% are considered ready biodegradation (RB) and those with a BOD value of less than 60% are considered not ready biodegradation (NRB). Of the total molecules, 1055 were used for the feature selection process and the remaining 670 were used to perform an external validation process.

### 2.5. Random Experiments Methods for Validation

In order to evaluate the risk of a random correlation in a subset of selected molecular descriptors, an fs-randomization (feature selection randomization) technique was used. This method consists of randomly selecting a set of descriptors (with the same cardinality of the subset selected by a specific technique) from the original set of features. With these descriptors and the property original values, a new model is generated with the same experimental criteria that were used to obtain the final QSAR model. Finally, the percentage of correctly classified cases (%CC) and the Matthews Correlation Coefficient (MCC) are reported. This procedure is executed a considerable number of times in order to obtain a distribution of values with statistical significance.

A similar procedure was performed to evaluate the random correlation of the final QSAR model inferred from a set of descriptors using y-randomization [52]. This technique randomly reorders the property values (y-variable) and leaves the selected molecular descriptors intact. In this way, a new model is inferred using the molecular descriptors of the final QSAR model and under the same experimental conditions but with the reordered values of the property. Like fs-randomization, this process is repeated a significant number of times, reporting the percentage of correctly classified cases (% CC) and the Matthews Correlation Coefficient (MCC) each time.

## 3. Results and Discussion

In this section, details of the modifications made to the two-phase method developed by Soto et al. will be provided [38]. Also, the MoDeSuS functionalities will be explained and finally a case study in the context of classification problems for QSAR modelling will be presented in order to illustrate the software application in pharmacology.

### 3.1. MoDeSuS Tool

**Figure 3:**
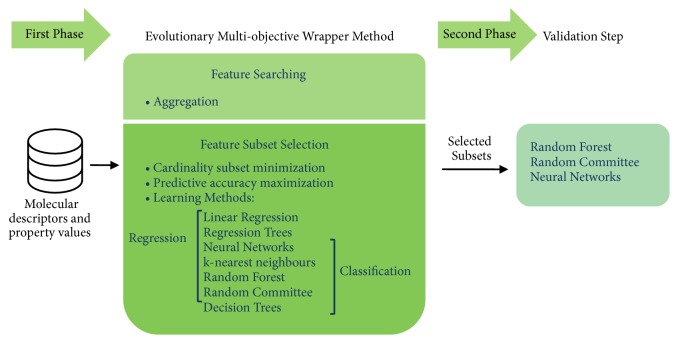
MoDeSuS two-phase feature selection methodology.

As mentioned above, MoDeSuS relies on the methodology presented in [38] that implements a multiobjective optimization based on two phases with some modifications introduced to contemplate classification problems and update the machine learning methods used.  Figure 3 shows an overview of MoDeSuS two-phase methodology.

In the* first phase*, two significant changes were made. One of them was to introduce a modification in the aggregation formula to allow dealing with classification problems. In this sense, the formula of (1) is still used, but in this case the objective function F2 changes depending on whether it is a regression or classification problem. That is, in the context of regression, F2 continues to estimate the mean square error of prediction, but in the classification context, F2 will now compute the percentage of cases that were not correctly classified by the predictor. The other change made is related to the machine learning methods provided. It can be seen in Figure 3 that the tool provides a wide variety of methods for both regression and classification problems. Finally, in the* second phase *an external validation of the selected subsets is carried out using one of three possible machine learning methods: Random Forest, Random Committee, and Neural Networks.

### 3.2. Software Functionalities

**Figure 4:**
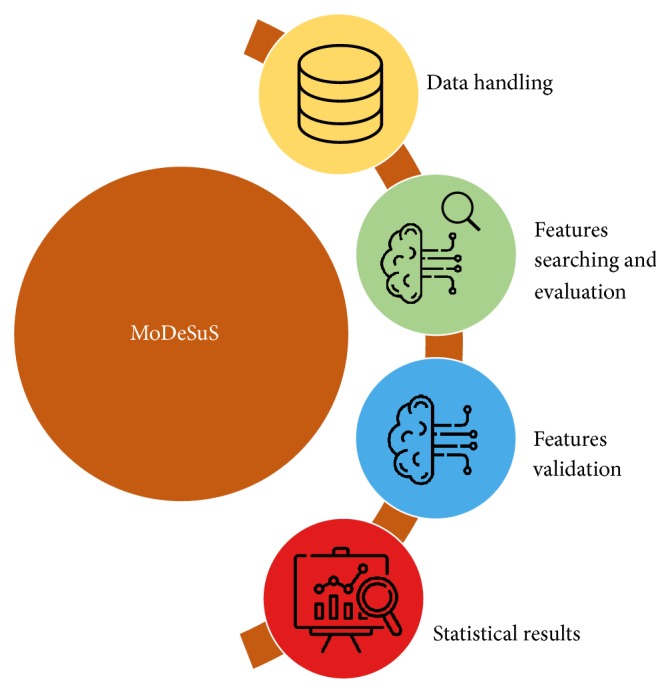
MoDeSuS functionalities.

MoDeSuS provides a graphical interface allowing the user to use the software without needing to know specific details of the code or of the different methods applied and a variety of features that will be explained below and can be summarized in Figure 4.

*Data handling* contains the functions for data loading. The input data must be in the CSV file format. The size of the entered data is verified and the computation results of each phase can be saved and restored later.

*Feature searching and evaluation* is the module that carries out the multiobjective evolutionary wrapping method of the first phase. The user can configure all the parameters of this phase, for example, the parameters of the evolutionary algorithm, and also select the automated learning method with which the different subsets of descriptors will be evaluated.

*Feature validation* provides the functionalities to configure parameters and execute the second phase of the method.

*Statistical results* are the module through which the user visualizes and interacts with the results obtained from the second phase. It is possible to access the information of the descriptors subsets that have been selected as well as different statistical metrics depending on the problem being addressed (regression or classification). In addition, the option of filtering the data is available, that is, choosing a specific subset and saving it.

### 3.3. Case Study and Performance Assessment

**Figure 5:**
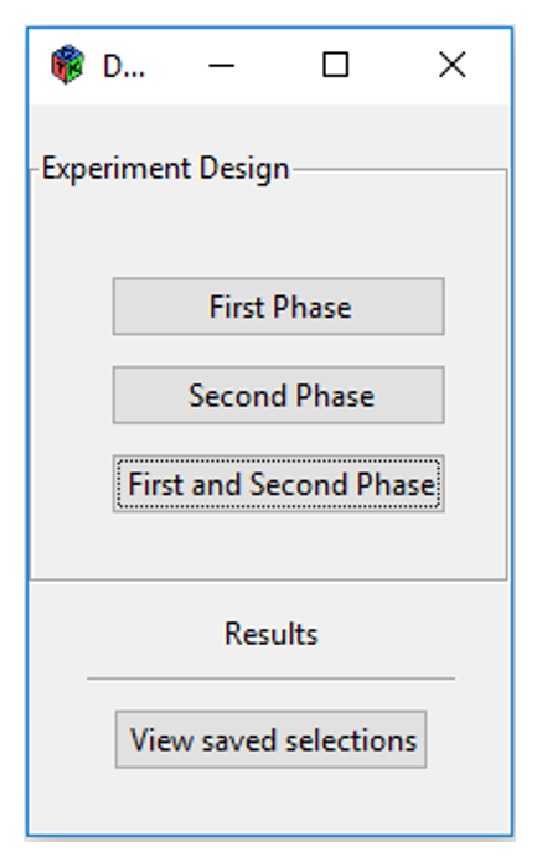
MoDeSuS initial view.

In this section, a case study in the context of classification problems to illustrate in detail the use of MoDeSuS in pharmacology will be explained. The property under study corresponds to the ready biodegradation of chemical compounds. When executing the tool several options will be available (Figure 5). There is the possibility of executing each phase separately or executing both phases sequentially. This last option was chosen to carry out our study. The results are shown immediately or can be consulted later with the option provided for that purpose.

**Figure 6:**
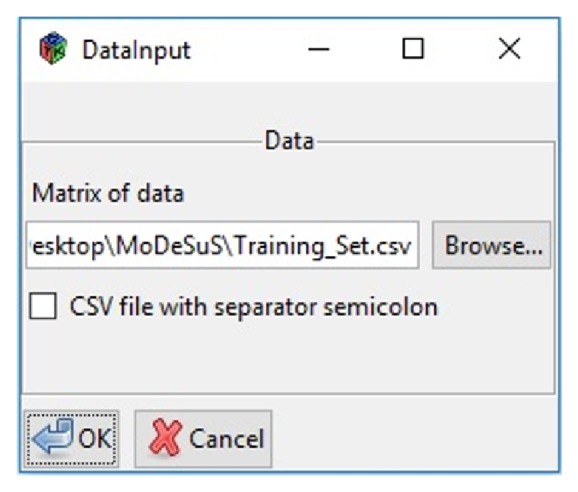
MoDeSuS data loading.

When choosing the “First and Second Phase” option, a data loading window will be displayed (Figure 6). In this window, a file in CSV format containing the values and names of the molecular descriptors and the class labels for ready biodegradation was loaded. In addition, it must be specified if the CSV file separator is a comma or a semicolon.

**Figure 7:**
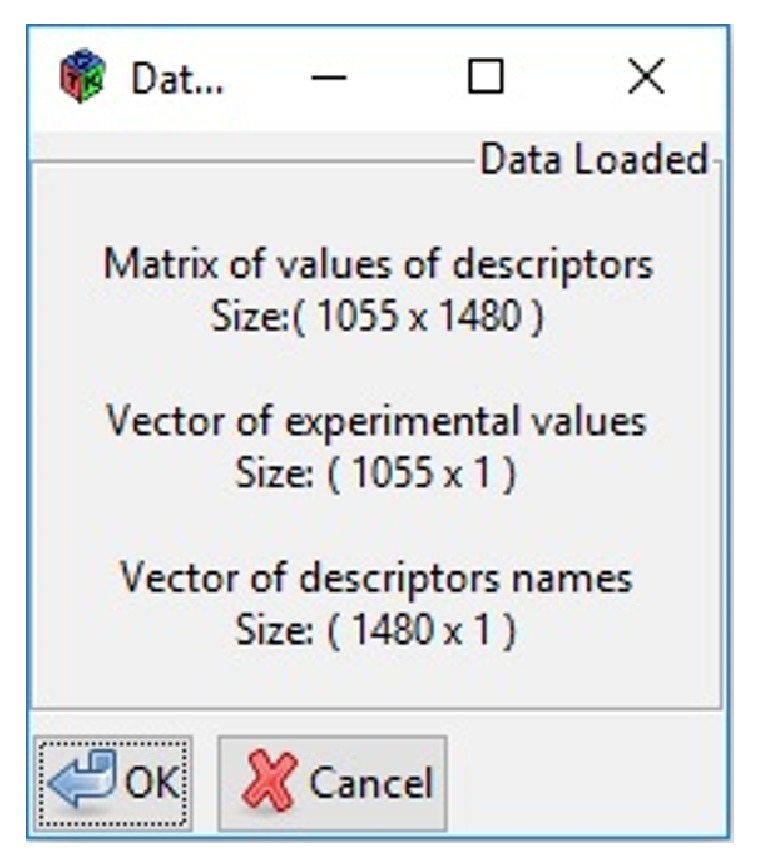
MoDeSuS data loading verification.

After data loading, another window will be displayed (Figure 7) in order to verify the data size. In our study a database with 1055 compounds and 1480 molecular descriptors was used.

**Figure 8:**
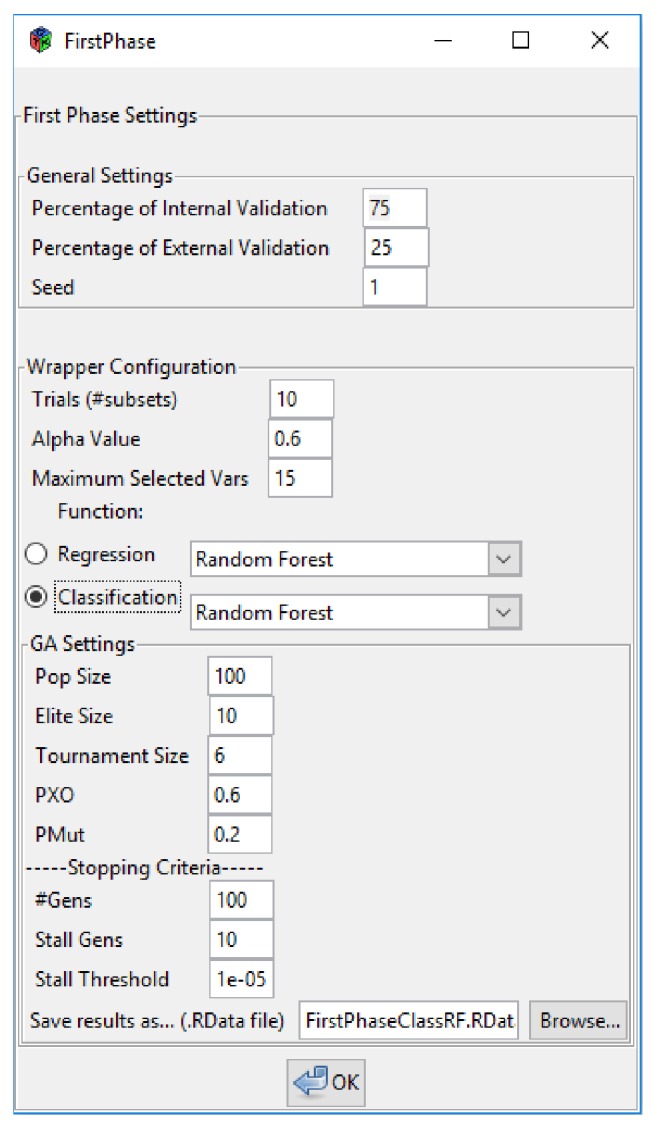
MoDeSuS first phase.

When verifying that the data size is correct, the execution continues displaying the first phase parameters configuration window (Figure 8). These parameters will have default values that the user can change according to their needs. In case there is an error in the loaded data, it is possible to go back and carry out the data loading again. It is important to mention that before starting the execution of the search algorithm, the tool performs an analysis of the data eliminating those variables with constant value. In this way, the number of descriptors loaded initially can be reduced before executing the first phase.

In Figure 8, it can be seen the parameters configuration used for our experiment. This window consists of three main sections. In the General Settings section, it is possible to define the* percentage of internal validation,* the* percentage of external validation*, and the* seed*. These values are necessary to determine the data partitions that will be used in each phase: one set of data for internal validation (to be used in the first phase) and another set of data remaining for external validation (to be used in the second phase). The seed value can be changed to perform different data partitions.

In the Wrapper Configuration section, it is possible to configure all the parameters that the wrapping method needs to perform the search. In this sense, the* trials* parameter refers to the number of final subsets that will be generated, the* alpha value *refers to the value “*α*” that is part of the aggregation formula (see (1)) and that will establish the weight that will be given to each objective function, and the* maximum cardinality* of the descriptors subsets. In addition, there are two lists of machine learning methods for regression and classification. The method selected will be the one used to estimate the predictive capacity of the descriptors subsets.

**Figure 9:**
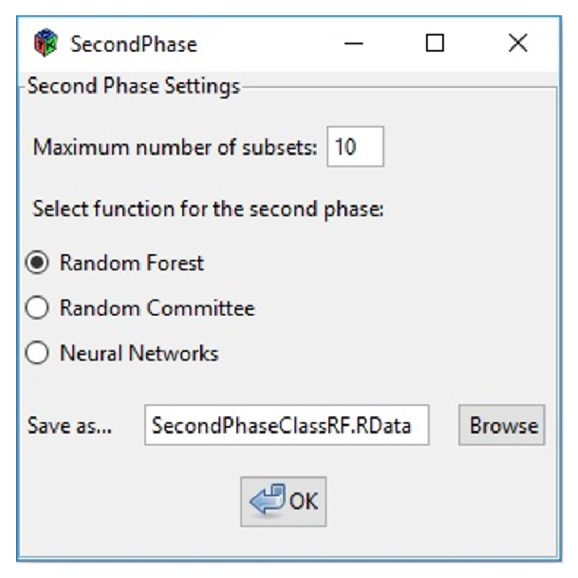
MoDeSuS second phase.

In GA Settings section, it is possible to configure all the parameters associated with the evolutionary method. It is possible to determine the* size of the population* (the number of individuals participating in each generation of the algorithm), the* size of the elite* (the number of individuals that are preserved from one generation to the next), the* size of the tournament* (the number of individuals competing in a tournament), the probability that two individuals will recombine (*PXO*), and the probability that an individual will suffer a mutation (*PMut*). In addition, stop criteria are set, such as the maximum number of generations that can be executed (*#Gens*), the maximum number of consecutive generations during which the average fitness of the population may not show significant improvements (*Stall Gens*), and the threshold (*Stall Threshold*) that defines the minimum (positive) difference that must exist between the average fitness of the population of one generation and the next to consider that there was improvement. Finally, the file name for saving the search results must be defined. This file will be used later for the execution of the second phase (Figure 9) whose window becomes visible once the execution of the first phase has finished.

In Figure 9 the parameters used in our case study are shown. It is possible to choose the number of subsets to evaluate and the machine learning method. This external validation will be carried out with the percentage of data that was set in the window of the first phase (Figure 8). Finally, the file name for saving the evaluation results must be defined. This file will be used for the results view.

**Figure 10:**
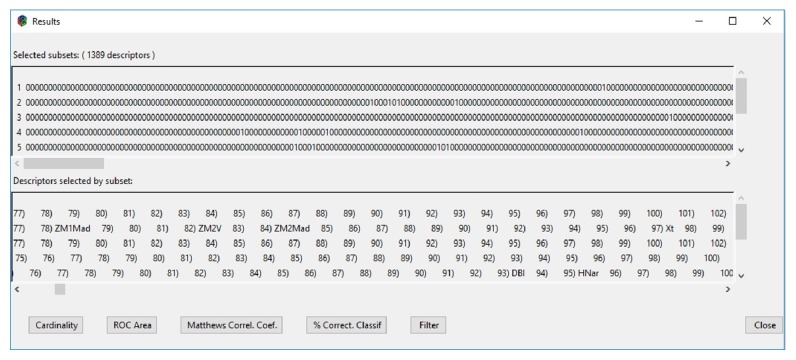
MoDeSuS results view.

Once the execution of the second phase is finished, the results window will be displayed (Figure 10). In this window two different views of the selected subsets and different options of statistical metrics can be seen that can be visualized through the graphics. These metrics are as follows: the percentage of cases correctly classified, the Average Receiver Operating Characteristic (ROC), the Matthews Correlation Coefficient (MCC), and the cardinality. In this case, Figure 10 shows the results for the 10 subsets of descriptors that were selected for the ready biodegradation property.

**Figure 11:**
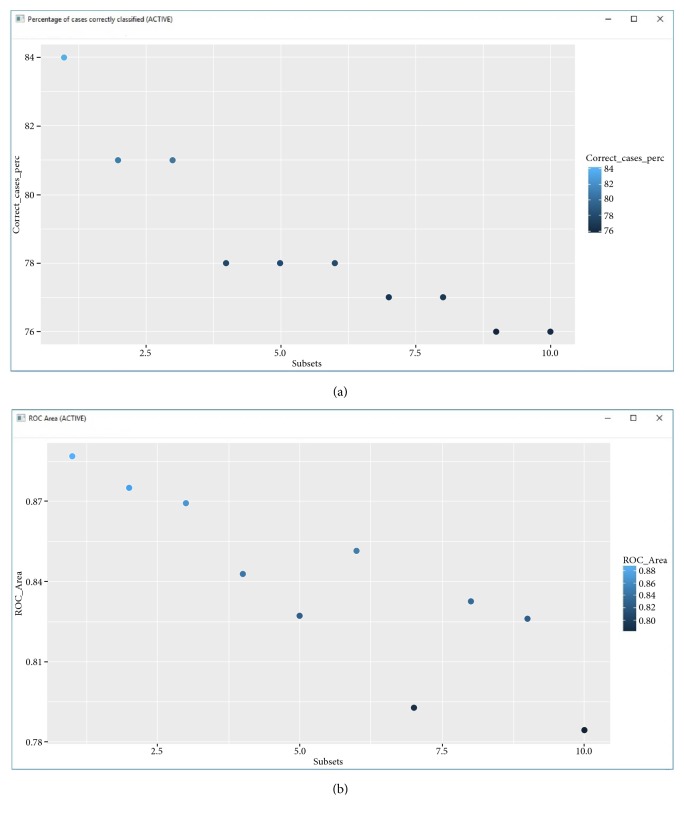
MoDeSuS graphics: (a) Percentage of Cases Correctly Classified (%CC) and (b) Average Receiver Operating Characteristic (ROC).

By pressing each button corresponding to the different statistical metrics, each of the graphs will be displayed.  Figure 11 shows the graphs corresponding to the percentage of cases correctly classified (% CC) and the Average Receiver Operating Characteristic (ROC), respectively. For our study we decided to stay with the three best subsets of the 10 reported by MoDeSuS and with them perform a final external validation stage with 670 compounds.  Table 1 shows a summary of metrics for the three subsets with higher accuracy predictive.

With the three subsets reported in Table 1, an external validation process using the 670 compounds for testing was performed using Weka [53] in the following way: for each subset (Subset_1C, Subset_2C, and Subset_3C) 10 runs were made using Random Forest (the same machine learning method that was used during the descriptor selection stage) with different seed values. The predictive accuracy for each subset can be seen in Table 2. The best performance was obtained for Subset_1C (shown in bold in Table 2) with 87% of correctly classified cases, a Matthews Correlation Coefficient of 0.67, a precision value of 0.87, and a recall value of 0.87 on average.

Based on the results shown in Table 2, it is recommended to use the model learned from the descriptors of Subset_1C for future predictions on new data. The performance achieved by this model is slightly lower than that reported by [50] (precision: 0.94 and recall: 0.81). In this sense, it is important to clarify that the performance obtained by [50] was achieved by filtering 13% of the external validation compounds using applicability domain techniques and constructing the model through consensus with a number of descriptors significantly greater than that of Subset_1C. In this sense, it is important to highlight that the specific objective of this case study is to illustrate the application of MoDeSuS on a dataset already used, in order to find a set of molecular descriptors that generate models with high predictive precision and with a low number of descriptors, both conditions that are defined in the tool through the fitness function of the evolutionary algorithm.

### 3.4. Random Experiments

In this section, two experiments will be presented in order to evaluate the risk of a random correlation in both the final descriptors subset chosen (Subset_1C) and in the final QSAR model inferred from these molecular descriptors. In this sense, the first aspect to evaluate is whether the Subset_1C selected by MoDeSuS has a significantly high predictive accuracy than other subsets of descriptors (of the same cardinality) randomly selected. Then, in a second instance, the final QSAR model is evaluated in order to ensure that it is not classifying compounds randomly.

In the first instance, a feature selection randomization (fs-randomization) was carried out in the following way: a thousand combinations of fifteen descriptors were randomly selected from the initial set of 1480 molecular descriptors. Then, for each random subset, a new QSAR model was learned under the same experimental conditions as the final QSAR model, finally reporting the %CC and MCC values.  Table 3 shows the results obtained. For the case of %CC, the mode is 79.85 with a variance value of 8.81. In addition, 99% of the %CC values obtained by fs-randomization are below 86.71 and the final QSAR model inferred from the descriptors obtained by MoDeSuS (Subset_1C) reported 87% of correctly classified cases, showing a significantly higher performance than the subsets random. In the case of MCC, the mode is 0.53, the variance is 0.01, and 99% of the values reported by fs-randomization are below 0.66. The MCC value reported by the final QSAR model was 0.67, again overcoming the predictive capacity of the random subsets.

As a next step, a y-randomization experiment has been executed. This technique is probably considered as the most powerful form of validation to evaluate the risk of chance correlation in QSAR models [54] and also when combined with the experimentation fs-randomization allows ensuring the reliability of QSAR models [53]. For this purpose, the values of the property are mixed randomly (both in the training and in the external validation set) leaving intact the Subset_1C descriptors obtained by MoDeSuS. This procedure is repeated a thousand times, and each time a new QSAR model is generated following the same experimental conditions as the final QSAR model and the values of % CC and MCC are reported. In Table 3 the metrics obtained are reported. For the % CC, the mode is 63.73, the variance is 4.10, and 99% of the values reported by y-randomization are less than 68.05. In the case of MCC, the mode is 0.03, the variance of 0.001 and 99% of the values reported by the experiment is less than 0.09. In this sense, it is possible to observe that the performance obtained by the model inferred from the descriptors of Subset_1C is significantly higher than the yields obtained through y-randomization. Consequently, according to the results of the two experiments, it is possible to discard safely the risk of correlation by chance in the final QSAR model learned with the descriptors selected by MoDeSuS.

## 4. Conclusions

In this paper, a novel software tool for selection of molecular descriptors subsets in QSAR modelling is presented. This new feature selection tool, named MoDeSuS, was designed in order to address this task for regression and classification problems. The computational methodology behind MoDeSuS is organized as a two-phase procedure. The first one makes use of a multiobjective evolutionary technique that identifies promising subsets of molecular descriptors following a wrapper technique. The second phase complements the first one and it enables refining and improving the confidence in the chosen subsets of descriptors by using complex machine learning methods: Random Forest and Random Committee. Additionally, several visualization modes for the different metrics reported for classification and regression modelling are included in the software.

MoDeSuS facilities and functionalities had been illustrated by using the tool in a cases study that constitutes an example for classification QSAR modelling, where the estimated property corresponds to ready biodegradation of chemical compounds. Comparisons with the performance achieved by others QSAR studies had been discussed, showing the potentially and usefulness of this novel software. For that reason, we think that MoDeSuS can constitute a valuable tool for QSAR modelling practitioners, helping to reduce time and money costs in drug development projects.

As future work, we plan to extend our software tool for considering the applicability domain of the QSAR models, evolved from the different subsets of selected molecular descriptors recommended by MoDeSuS, as an additional performance metric. The applicability domain estimation is a key issue in QSAR modelling, because the generalizability of the models depends on it. This goal can be achieved by integrating, in the fitness function of the evolutionary algorithm, information about the applicability domain of the QSAR models generated by each subset of selected molecular descriptors explored during the first phase of MoDeSuS. In this way, the feature selection will not only produce accurate and interpretable QSAR models, but also ensure enhanced generalizability on new data, deriving in more reliable predictions.

## Figures and Tables

**Table 1:** Performances of the three subsets with higher accuracy predictive obtained by using MoDeSuS. The percentage of cases correctly classified (%CC), the Average Receiver Operating Characteristic (ROC), the Matthews Correlation Coefficient (MCC), and the cardinality are reported.

Metrics	Subset_1C	Subset_2C	Subset_3C
%CC	84	81	81
ROC	0.89	0.88	0.87
MCC	0.7	0.66	0.64
Cardinality	15	15	15

**Table 2:** Predictive accuracy of external validation process over subsets 1C, 2C, and 3C by using Weka. The percentage of cases correctly classifies (%CC), the Matthews Correlation Coefficient (MCC), precision (PR), and recall (RC) values is reported.

	*Subset_1C*				*Subset_2C*				*Subset_3C*			
Run	%CC	MCC	PR	RC	%CC	MCC	PR	RC	%CC	MCC	PR	RC
1	86.12	0.65	0.86	0.86	83.28	0.57	0.83	0.83	84.77	0.61	0.84	0.85

2	86.86	0.67	0.87	0.87	83.43	0.57	0.83	0.83	85.37	0.63	0.85	0.85

3	86.41	0.66	0.86	0.86	83.13	0.57	0.83	0.83	85.52	0.63	0.85	0.86

4	86.86	0.67	0.86	0.87	82.53	0.55	0.82	0.83	84.77	0.61	0.84	0.85

5	87.46	0.68	0.87	0.88	83.43	0.56	0.83	0.83	84.62	0.61	0.84	0.84

6	87.31	0.68	0.87	0.87	84.47	0.6	0.84	0.85	85.22	0.63	0.85	0.85

7	87.46	0.68	0.87	0.88	83.43	0.58	0.83	0.83	85.07	0.62	0.85	0.85

8	85.97	0.65	0.86	0.86	82.98	0.57	0.83	0.83	85.67	0.64	0.85	0.86

9	87.31	0.68	0.87	0.87	82.23	0.54	0.82	0.82	84.62	0.61	0.84	0.85

10	87.61	0.69	0.87	0.88	82.23	0.54	0.82	0.82	85.07	0.62	0.85	0.85

Avg.	**87.00**	**0.67**	**0.87**	**0.87**	83.00	0.57	0.83	0.83	85.00	0.62	0.85	0.85

**Table 3:** Statistical results for *fs-randomization* and *y-randomization* and performance of the model inferred from descriptors of Subset_1C (Model_Subset1C_). For each case, for the percentage of cases correctly classified (%CC) and the Matthews Correlation Coefficient (MCC), three metrics are reported: mode, variance, and percentile_99_.

*fs – randomization*				*Model*_*Subset*1*C*_
	Mode	Variance	Perc_(99)_	

%CC	79.85	8.81	86.71	87
MCC	0.53	0.01	0.66	0.67

*y – randomization*				*Model*_*Subset*1*C*_
	Mode	Variance	Perc_(99)_	

%CC	63.73	4.10	68.05	87
MCC	0.03	0.001	0.09	0.67

## Data Availability

The data used to support the findings of this study are available from the corresponding author upon request.
